# In situ vaccination using unique TLR9 ligand K3-SPG induces long-lasting systemic immune response and synergizes with systemic and local immunotherapy

**DOI:** 10.1038/s41598-022-05702-0

**Published:** 2022-02-08

**Authors:** Hirokazu Okada, Ken Takahashi, Hiroaki Yaku, Kouji Kobiyama, Keiko Iwaisako, Xiangdong Zhao, Masahiro Shiokawa, Norimitsu Uza, Yuzo Kodama, Ken J. Ishii, Hiroshi Seno

**Affiliations:** 1 Department of Gastroenterology and Hepatology, Graduate School of Medicine, Kyoto University, 54-Syogoin Kawara-cho, Sakyoku, Kyoto, 606-8507 Japan; 2 Division of Vaccine Science, The Institute of Medical Science, The University of Tokyo, 4-6-1 Shirokanedai, Minato-ku, Tokyo, 108-8639 Japan; 3 Department of Medical Life Systems, Faculty of Life and Medical Sciences, Doshisha University, 1-3 Tatara Miyakodani, Kyotanabe-shi, 610-0394 Japan; 4 Division of HBP Surgery and Transplantation, Department of Surgery, Kyoto University, 54-Shogoin Kawahara-cho, Sakyoku, Kyoto, 606-8507 Japan; 5 Department of Gastroenterology and Hepatology, Graduate School of Medicine, Kobe University, 7-5-1 Kusunoki-cho, Chuo-ku, Kobe, 650-0017 Japan

**Keywords:** Tumour immunology, Immunotherapy, Innate immunity

## Abstract

Although checkpoint inhibitors (CPIs) have changed the paradigm of cancer therapy, low response rates and serious systemic adverse events remain challenging. In situ vaccine (ISV), intratumoral injection of immunomodulators that stimulate innate immunity at the tumor site, allows for the development of vaccines in patients themselves. K3-SPG, a second-generation nanoparticulate Toll-like receptor 9 (TLR9) ligand consisting of K-type CpG oligodeoxynucleotide (ODN) wrapped with SPG (schizophyllan), integrates the best of conventional CpG ODNs, making it an ideal cancer immunotherapy adjuvant. Focusing on clinical feasibility for pancreaticobiliary and gastrointestinal cancers, we investigated the antitumor activity of K3-SPG-ISV in preclinical models of pancreatic ductal adenocarcinoma (PDAC) and colorectal cancer (CRC). K3-SPG-ISV suppressed tumor growth more potently than K3-ISV or K3-SPG intravenous injections, prolonged survival, and enhanced the antitumor effect of CPIs. Notably, in PDAC model, K3-SPG-ISV alone induced systemic antitumor effect and immunological memory. ISV combination of K3-SPG and agonistic CD40 antibody further enhanced the antitumor effect. Our results imply that K3-SPG-based ISV can be applied as monotherapy or combined with CPIs to improve their response rate or, conversely, with CPI-free local immunotherapy to avoid CPI-related adverse events. In either strategy, the potency of K3-SPG-based ISV would provide the rationale for its clinical application to puncturable pancreaticobiliary and gastrointestinal malignancies.

## Introduction

The field of cancer immunotherapy is rapidly evolving. The great clinical achievements of antagonizing monoclonal antibodies against cytotoxic T-lymphocyte-associated protein 4 (CTLA-4) and programmed cell death 1 (PD-1), called checkpoint inhibitors (CPIs), have clearly proven that the immune system is capable of eradicating tumor cells, validating the concept of harnessing the patient’s own immune system to control cancer^1^. However, only a minority of patients respond to these CPIs^2,3^, and the serious adverse events due to autoimmune mechanisms limit their use^4^. This highlights the unmet medical need for new immunotherapies.

CpG oligodeoxynucleotide (ODN), a ligand for Toll-like receptor 9 (TLR9), stimulates dendritic cells (DCs) to produce cytokines such as type I interferon (IFN) and interleukin (IL)-12, which induce a Th1-type response characterized by IFN-γ production. This is a critical step in the activation of naïve T cells to functional antitumor CD8 T cells called cytotoxic T lymphocytes (CTLs), central players in cancer immunity^5–7^. Accordingly, a number of preclinical and clinical studies have evaluated the antitumor activity of CpG ODNs by various routes of administration, some of which have shown promising results^8^. CpG ODNs are categorized into A/D-, B/K-, C-, and P-types. A/D- and P-type CpG ODNs are potent type I IFN inducers, because the higher structure formed by base paring between their palindromic sequences causes their localization to early endosome that is prerequisites for robust type I IFN induction^9–11^. A general challenge for their clinical application is that their higher structure results in aggregation, while CpG-A ODN designated as CMP-001 that is packaged in a virus-like particle has been developed and tested in clinical trials^12,13^. B/K- and C-type CpG ODNs are also applied for clinical use, and among them, the B/K type has been the most extensively studied^9,14,15^. However, these types of ODNs are weaker type I IFN inducers than A/D- and P-type CpG ODNs^16^.

We recently developed a second-generation TLR9 agonist designated as K3-SPG, which is a nanoparticulate K-type CpG ODN (K3) wrapped with the nonagonistic Dectin-1 ligand schizophyllan (SPG)^9^. It forms a completely solubilized higher order nano-sized particle. This structure enables it to gain the function of A/D type CpG ODN without losing that of K type. Consequently, K3-SPG overcomes the limitations of conventional CpG ODNs including K3, in that it is a robust inducer of type I IFN and Th1 type cytokines without aggregation^9^. Indeed, in sharp contrast to K3, K3-SPG is a potent Th1-type adjuvant for CTL induction in mice and nonhuman primates (NHPs)^9,17–20^. Importantly, intravenous (iv) injection of K3-SPG in transplantable mouse tumor model resulted in the accumulation of K3-SPG in the tumor microenvironment (TME), induced tumor-specific CTLs, and suppressed tumor growth. The underlying mechanism involved the induction of Th1-type immune response (i.e., type I IFN and IL-12) as well as immunogenic tumor cell death (ICD)^21^. This unique property of K3-SPG makes it an ideal immunomodulator for cancer vaccines.

In situ vaccines (ISVs), intratumoral injections of immunostimulatory adjuvants, activate the innate immunity directly at the tumor site, where the tumor itself provides the antigens for vaccine, thereby inducing potent systemic as well as memory adaptive immunity^22,23^. ISV has several unique advantages^22,23^. First, unlike standard methods, ISV generates cancer vaccines in vivo without any process of identification and preparation of tumor antigens. This feature contrasts with the laborious, expensive, and time-consuming efforts in personalized neoantigen vaccines that require multiple steps of sampling tumor tissues, sequencing tumor genome, prediction, and identification of putative antigens in the context of individual human leukocyte antigen types, and the in vitro generation of these antigens^24^. Furthermore, while the standard vaccines target only a single or a limited number of antigens, ISV can theoretically induce CTLs with different antigen specificities to obtain a complete antigen repertoire, minimizing the risk of immune escape by antigen loss. Second, local activation of innate immunity at the tumor site, which induces type I IFNs and Th1 type cytokines, transforms the immunosuppressive TME into immunosupportive tissue. Third, systemic and long-lasting memory responses in the setting of localized treatment are expected. Finally, compared with the systemic administration of immunomodulators, systemic adverse effects are expected to be minimized despite high concentrations at the tumor site.

Pancreatic ductal adenocarcinoma (PDAC) and colorectal cancer (CRC) are among the most aggressive malignancies with increasing incidence in recent years^25^, for which conventional chemotherapies and current immunotherapies are not satisfactorily effective^26,27^. From a clinical viewpoint, ISV is a feasible strategy for pancreaticobiliary and gastrointestinal cancers because endoscopic ultrasound (EUS)- or endoscopy-guided tumor puncture is widely applied in clinical settings not only for diagnostic but also for therapeutic purposes^28,29^. In the present study, we sought to clarify the antitumor activity of K3-SPG-ISV using preclinical models of PDAC and CRC. Our results revealed that K3-SPG-ISV monotherapy sufficiently induced systemic and long-lasting memory responses and that it acted synergistically with both systemic administration of CPIs and local administration of a CD40 agonist, another innate immune stimulator^30^, establishing the proof of concept for its clinical application in these intractable cancers, for which tumor puncture is a routine clinical technique.

## Results

### K3-SPG induces type I IFN and Th1 type immune response more potently than conventional TLR9 ligands

**Figure 1:**
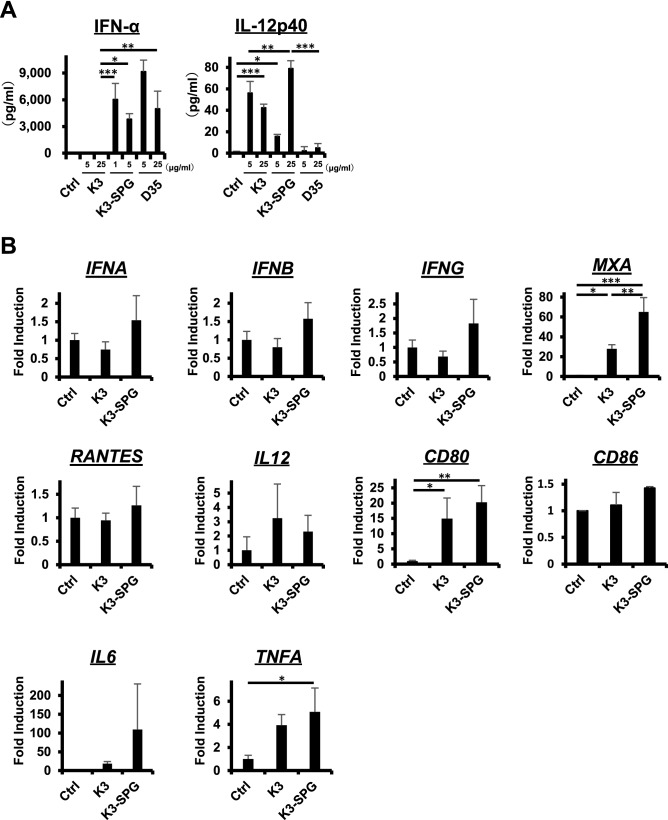
K3-SPG induces type I IFN and Th1 type immune response more potently than conventional TLR9 ligands. (**A**) (Left panel) Production of IFN-α by human PBMC stimulated with K3 (5, 25 μg/mL), K3-SPG (1, 5 μg/mL), and D35 (5, 25 μg/mL) for 24 h was measured by ELISA. (Right panel) Production of IL-12p40 by human PBMC stimulated with K3 (5, 25 μg/mL), K3-SPG (5, 25 μg/mL), and D35 (5, 25 μg/mL) for 24 h was measured by ELISA. (**B**) The relative expression levels of *IFNA, IFNB, IFNG, MXA, RANTES, IL12, CD80, CD86, IL6* and *TNFA* mRNA induced by K3 (5 μg/mL) or K3-SPG (1 μg/mL) were measured by quantitative real-time PCR. The results were normalized to the expression of GAPDH. Data are representative of two independent experiments with similar results. Error bars represent the mean ± SEM. Statistically significant differences were measured by one-way ANOVA followed by the Tukey–Kramer test. *p < 0.05; **p < 0.01; ***p < 0.001.

First, the potential of K3-SPG as an effective vaccine adjuvant was compared with two conventional TLR9 ligands, K3 (K-type CpG ODN) and D35 (D-type CpG ODN). Type I IFN plays an essential role in the induction of cancer immunity. K3 is a weak type I IFN inducer, while D35 is a robust type I IFN inducer that is, however, difficult to translate into clinical application due to aggregation. In an in vitro human peripheral blood mononuclear cell (PBMC) experiment, enzyme-linked immunosorbent assay (ELISA) analysis showed that K3-SPG produced higher amounts of IFN-α than K3, which was comparable to that produced by D35 (Fig. 1A, left panel). IL-12, which is also an essential cytokine for antitumor immunity that skews to Th1 response, was produced by K3-SPG as did K3, while D35 only marginally produced it (Fig. 1A, right panel). Expression analysis of mRNA revealed that K3-SPG induced type I and II IFNs and their inducible genes (*IFNA*, *IFNB*, *IFNG*, *MXA*, and *RANTES*), DC activation markers (*CD80* and *CD86*), and inflammatory cytokines (*IL6* and *TNFA*) to a greater extent than K3, although not all genes with statistical significance. IL-12 induction was slightly stronger with K3 than K3-SPG (Fig. 1B). The observed discrepancy in the cytokine levels between ELISA and real-time PCR was presumably attributed to the difference in the degradation rates of protein and mRNA.

Consistent with these human PBMC results, in mouse splenocyte experiments, K3-SPG showed higher production of IFN-α and IL-12 than K3 in ELISA (Supplementary Fig. 1A) and the upregulation of IFNs and their related genes, DC activation markers, and inflammatory cytokines at mRNA expression levels (Supplementary Fig. 1B). These results suggest K3-SPG might be superior to other conventional CpG ODNs as a cancer vaccine adjuvant.

### K3-SPG-ISV induces intratumoral type I IFN and Th1 type immune response, suppresses tumor growth, and prolongs survival

**Figure 2:**
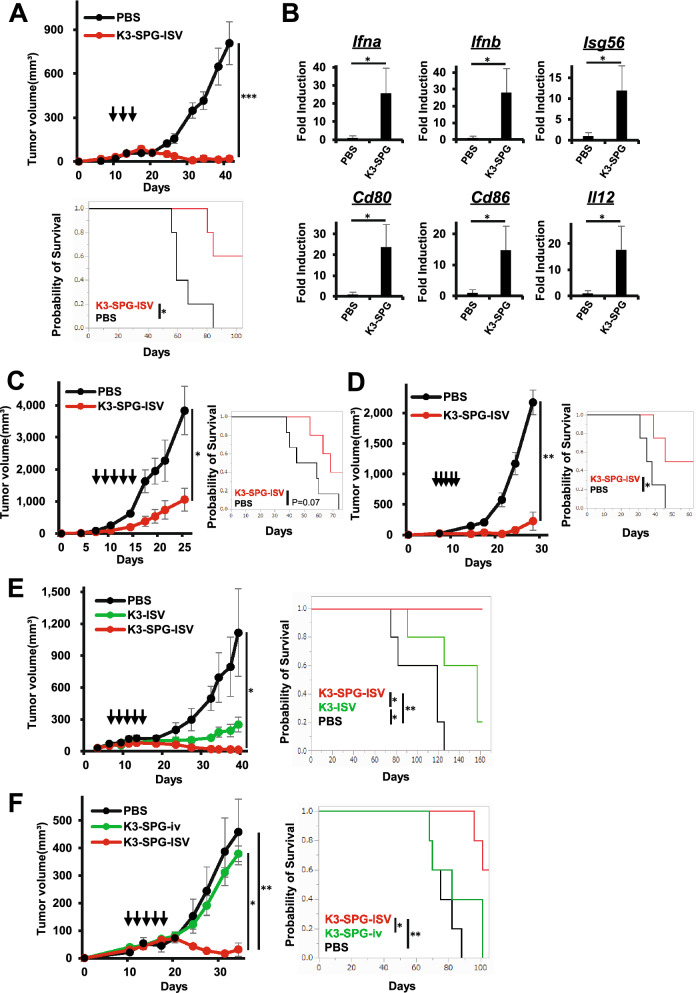
K3-SPG-ISV induces intratumoral type I IFN and Th1 type immune responses, suppresses tumor growth, and prolongs survival. (**A**) Tumor volume (upper panel) and survival rate (lower panel) were monitored among KPC-N-bearing mice treated with PBS or K3-SPG-ISV (10 μg) on days 10, 13, and 15 after tumor inoculation (n = 5). (**B**) Intratumoral mRNA expression levels of the indicated genes on day 19 in the same experimental protocol were measured by quantitative real-time PCR (n = 3). The results were normalized to the expression of 18S rRNA. (**C**) Tumor volume (left panel) and survival rate (right panel) were monitored among colon-26-bearing mice treated with PBS or K3-SPG-ISV (10 μg) on days 7, 9, 11, 13, and 15 after tumor inoculation (n = 6). (**D**) Tumor volume (left panel) and survival rate (right panel) were monitored among MC38-bearing mice treated with PBS or K3-SPG-ISV (10 μg) on days 7, 8, 9, 10, and 11 after tumor inoculation (n = 4). (**E**) Tumor volume (left panel) and survival rate (right panel) were monitored among KPC-N-bearing mice treated with PBS, K3-ISV (30 μg), or K3-SPG-ISV (10 μg) on days 7, 9, 11, 13, and 15 after tumor inoculation (n = 5). (**F**) Tumor volume (left panel) and survival rate (right panel) were monitored among KPC-N-bearing mice treated with PBS, K3-SPG-ISV (10 μg), or K3-SPG-iv (10 μg) on days 10, 12, 14, 17, and 18 after tumor inoculation (n = 5). The data are representative of three independent experiments with similar results. The arrows indicate the timing of therapy. Error bars represent the mean ± SEM. Statistically significant differences were measured by one-way ANOVA followed by Dunnett’s post hoc tests (panel A, B, C and D) and the Tukey–Kramer test (panel E and F). *p < 0.05; **p < 0.01; ***p < 0.001. Survival curves were analyzed using log-rank tests. iv, intravenous.

We investigated the antitumor effect of K3-SPG-ISV using a syngeneic pancreatic cancer model. C57BL/6 mice were challenged with 2 × 10^6^ mouse PDAC cell lines designated as KPC-N, established from Kras^LSL-G12D/+^, Trp53^LSL-R172H/+^, and Pdx1-Cre mice (KPC), injected into the left flank, followed by intratumoral injection of either phosphate-buffered saline (PBS) or K3-SPG from 10 days after tumor implantation. K3-SPG-ISV (i.e., K3-SPG-it) significantly suppressed tumor growth and prolonged survival compared to the control treatment (Fig. 2A). Recapitulating the results of in vitro stimulation experiments, as shown in Fig. 1, mRNA expression analysis of the TME after K3-SPG-ISV clearly revealed the upregulation of type I IFNs (*Ifna and Ifnb*), IFN-inducible gene (*Isg56*), IL-12, and DC activation markers (*Cd80 and Cd86*) (Fig. 2B). Tumor growth suppression and survival prolongation by K3-SPG-ISV were reproduced in another PDAC model using KPF-T cells (Supplementary Fig. 2) and two CRC models using colon-26- and MC38-bearing mice (Fig. 2C,D, respectively). These results indicated the generality of the antitumor effect of K3-SPG-ISV, with repeated experiment results showing an overall tendency of greater potency on PDAC than CRC models.

Next, we investigated the antitumor effect of K3-SPG-ISV compared to that of K3-ISV and intravenous administration of K3-SPG (K3-SPG-iv). K3-SPG-ISV significantly suppressed tumor growth and prolonged survival compared with K3-ISV (Fig. 2E). While our PDAC model of KPC-N-bearing mice was resistant to K3-SPG-iv, K3-SPG-ISV still remarkably suppressed tumor growth and prolonged survival compared with K3-SPG-iv (Fig. 2F). Taken together, these results indicate that K3-SPG-ISV has more potent therapeutic effects than conventional TLR9 ligand K3 or systemic administration of K3-SPG.

### Antitumor activity of K3-SPG-ISV potentiates the effect of checkpoint blockade therapies

**Figure 3:**
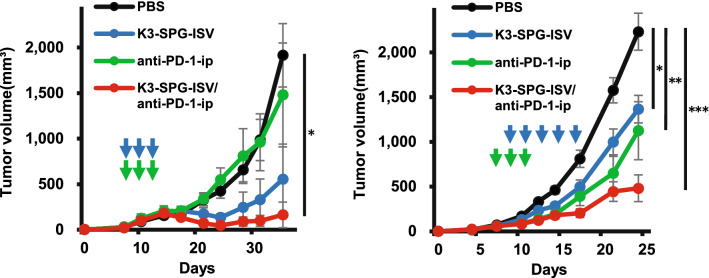
Antitumor activity of K3-SPG-ISV potentiates the effect of PD-1 blockade therapy. (Left panel) Tumor volume was monitored among KPC-N-bearing mice treated with PBS, K3-SPG-ISV (10 μg on days 8, 10, and 12), anti-PD-1-ip (100 μg on days 8, 10, and 12), or a combination of K3-SPG-ISV/anti-PD-1-ip (same dose and schedule as respective monotherapy) (n = 5). Data are representative of two independent experiments with similar results. (Right panel) Tumor volume was monitored among colon-26-bearing mice treated with PBS (n = 43), K3-SPG-ISV (10 μg on days 9, 11, 13, 15, and 17; n = 23), anti-PD-1-ip (100 μg on days 7, 9, and 11; n = 19), or a combination of K3-SPG-ISV/anti-PD-1-ip (same dose and schedule as respective monotherapy; n = 20). The pooled data of six independent experiments of the same protocol are presented. The arrows indicate the timing of therapy. Error bars represent the mean ± SEM. Statistically significant differences were measured by one-way ANOVA followed by the Tukey–Kramer test. *p < 0.05; **p < 0.01; ***p < 0.001. ip, intraperitoneal.

Systemic administration of CPIs, anti-PD-1 and anti-CTLA-4 antibodies, is the standard regimen for cancer immunotherapy. We evaluated the effect of K3-SPG-ISV on CPIs. In PDAC and CRC models, the anti-PD-1 antibody did not or partially suppressed tumor growth, respectively (Fig. 3). In both models, the combination of K3-SPG-ISV and PD-1 blockade enhanced the antitumor effect of each respective monotherapy (Fig. 3). Similarly, while anti-CTLA-4 antibody partially suppressed tumor growth in both models, the combination of K3-SPG-ISV and CTLA-4 blockade showed marked tumor growth inhibition, where the synergistic effect was clear particularly in the CRC model (Supplementary Fig. 3).

### K3-SPG-ISV induces immunological memory

**Figure 4:**
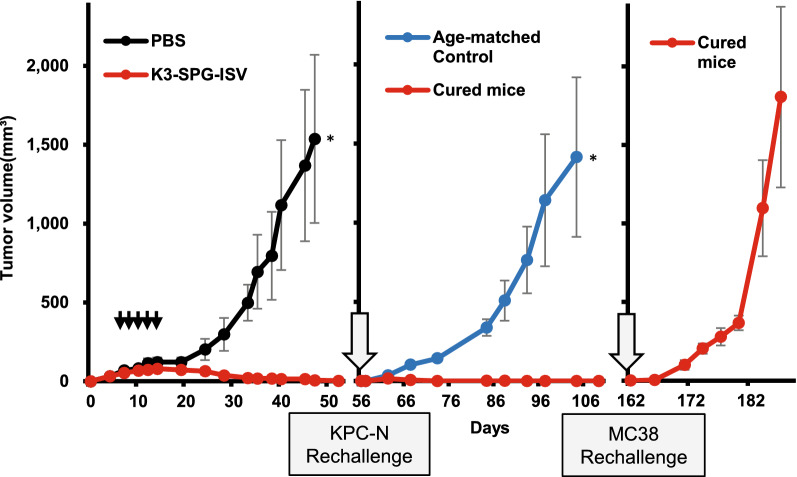
K3-SPG ISV induces immunological memory. KPC-N-bearing mice were treated with PBS or K3-SPG-ISV (10 μg) on days 7, 9, 11, 13, and 15 after tumor inoculation (n = 5). All the K3-SPG-ISV-treated mice were cured. On day 56, the cured mice were rechallenged with the second round of subcutaneous inoculation of 2 × 10^6^ KPC-N and age-matched naïve C57BL6 mice (n = 5) were subcutaneously inoculated with the same number of KPC-N as a control cohort. All cured mice rejected rechallenge with KPC-N. On day 162, they were subcutaneously challenged with 2 × 10^6^ MC38 cells. The tumor growth curves over the entire time course are presented. Data are representative of two independent experiments with similar protocols and results. The arrows indicate the timing of therapy. Error bars represent the mean ± SEM. Statistically significant differences were measured by one-way ANOVA with Dunnett’s post hoc test. *p < 0.05.

Motivated by the strong antitumor activity of K3-SPG-ISV, which frequently leads to the complete eradication of engrafted cells in the PDAC model, we investigated whether the mice cured after K3-SPG-ISV treatment developed immunological memory. PDAC mice that were cured after K3-SPG-ISV, followed by rechallenge with the same PDAC cells (i.e., KPC-N cells) 56 days after the first inoculation, completely rejected the replanted tumor cells, whereas all age-matched naïve control mice showed the growth of KPC-N cells (Fig. 4). Importantly, colon cancer MC38 cells, when inoculated into these cured mice that rejected replanted KPC-N cells, engrafted and expanded in all mice. These results clearly indicated that K3-SPG-ISV monotherapy induced immunological memory specific to PDAC cells. Although K3-SPG-ISV monotherapy failed to cure colon-26-bearing mice, the combination therapy of K3-SPG-ISV and PD-1 blockade cured half of the treated mice. All the mice cured by this combination rejected rechallenge with colon-26, while the same cells engrafted in the age-matched tumor-naïve control, indicating the establishment of immunological memory in these cured mice (Supplementary Fig. 4).

### K3-SPG-ISV induces a systemic antitumor effect that is dependent on CD8 T cells

**Figure 5:**
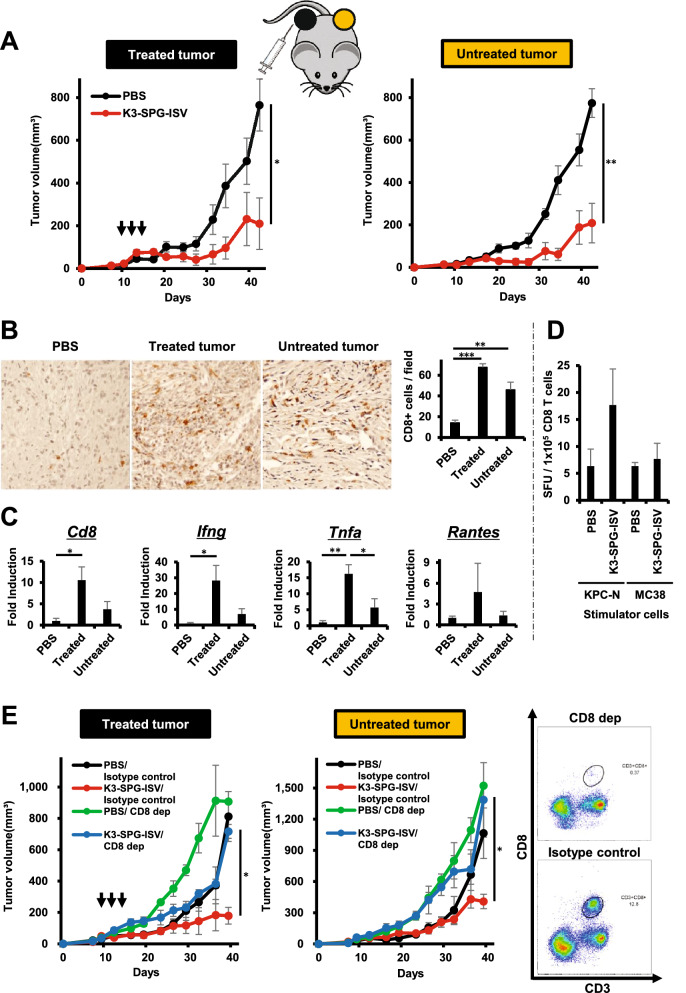
K3-SPG-ISV induces systemic antitumor effects that are dependent on CD8 T cells. (**A**) Mice bearing bilateral subcutaneous KPC-N tumors were treated with PBS or K3-SPG-ISV (10 μg) at one tumor site on days 10, 12, and 14 after tumor inoculation (n = 5). Tumor volumes on the treated side (left panel) and untreated side (right panel) are shown. (**B**) (Left panels) Mice bearing bilateral subcutaneous KPC-N tumors were treated with PBS or K3-SPG-ISV (10 μg) at one tumor site on days 9, 12, and 15 after tumor inoculation (n = 3), followed by the immunohistochemical detection of intratumoral CD8 T cells on day 19 (×200 magnification). (Right panel) The mean numbers of CD8 T cells in each cohort calculated from median values in the three random fields of each sample were displayed. (**C**) Intratumoral mRNA expression levels of the indicated genes on day 19 in the same experimental protocol were measured by quantitative real-time PCR (n = 3). (**D**) Mice bearing subcutaneous KPC-N tumor were treated with PBS or K3-SPG-ISV (10 μg) on days 9, 12, and 14 after tumor inoculation. On day 15, CD8 T cells were purified from spleens and cocultured with KPC-N or MC38 as stimulator cells. ELISPOT results of IFN-γ + CTLs in PBS- and K3-SPG-ISV-treated mice are shown (n = 3). E. Mice bearing bilateral subcutaneous KPC-N tumors were treated with PBS/isotype control, K3-SPG-ISV (10 μg)/isotype control, PBS/anti-CD8α, or K3-SPG-ISV (10 μg)/anti-CD8α at one tumor site (n = 5). K3-SPG-ISV was performed on days 9, 12, and 15 after tumor inoculation. Anti-CD8α was administered twice weekly for the duration of the experiment, starting on day 0. Tumor volume of the treated side (left panel) and untreated side (center panel) are shown. (Right panel) Flow cytometric analysis on day 39 demonstrated the depletion of CD8 T cells in the splenocytes of anti-CD8α-treated mice. The data are representative of three independent experiments with similar results. The arrows indicate the timing of therapy. Error bars represent the mean ± SEM. Statistically significant differences were measured by one-way ANOVA with Dunnett’s post-hoc test (panel A) and the Tukey–Kramer test (panel B, C and E). *p < 0.05; **p < 0.01. CD8 dep: CD8 depletion by anti-CD8α.

In addition to a durable memory response, another appealing point of immunotherapy is the potential for a systemic immune response called the abscopal effect. We investigated the abscopal effect induced by K3-SPG-ISV by evaluating tumor growth suppression on the opposite side of the vaccinated tumor. K3-SPG-ISV tested in mice bilaterally bearing KPC-N suppressed vaccinated tumors as well as untreated distant tumors, clearly demonstrating that K3-SPG-ISV induced the abscopal effect (Fig. 5A). A similar systemic antitumor effect was observed in the colon-26 model (Supplementary Fig. 5A).

Cancer vaccines aim to induce antitumor CD8 T cells that produce IFN-γ, a central player in cancer immunity. Immunohistochemistry analysis showed that K3-SPG-ISV increased CD8 T cell infiltration in both vaccinated and untreated tumors (Fig. 5B). Consistently, at the transcriptional level, upregulation of intratumoral *Cd8* and *Ifng* expression was observed in the tumor on both sides, although their induction was stronger in the vaccinated than in the untreated side (Fig. 5C). *Rantes*, an IFN-γ-inducible chemokine, and *TNFA* were also upregulated, although *Rantes* was marginally expressed in the untreated side (Fig. 5C). Enzyme-linked immunospot (ELISPOT) analysis showed that CD8 T cells purified from splenocytes of K3-SPG-ISV-treated KPC-N-bearing mice produced IFN-γ when cocultured with KPC-N as target cells but not with syngeneic control MC38 cells, indicating the induction of KPC-N-specific CTLs in the K3-SPG-ISV-treated mice (Fig. 5D). Under the condition that > 97% depletion of CD8 T cells was achieved in the splenocytes (Fig. 5E, right panel), CD8 T cell depletion canceled the antitumor activity of K3-SPG-ISV on both the vaccinated and untreated sides, indicating that ISV systemically suppressed tumor growth in a CD8 T cell dependent manner (Fig. 5E, left and center panels). CD8 T cell dependency of K3-SPG-ISV was also observed in the colon-26 model (Supplementary Fig. 5B).

### Combined ISV strategy incorporating agonistic CD40 antibody in K3-SPG enhances the antitumor effect

**Figure 6:**
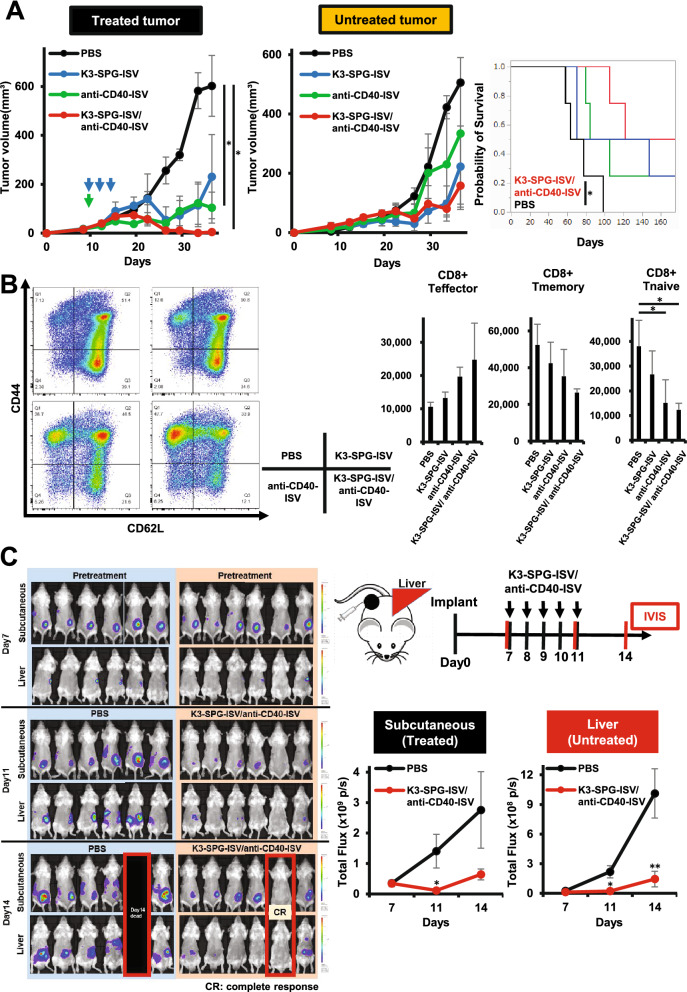
Combined ISV strategy incorporating agonistic CD40 antibody into K3-SPG enhances antitumor effect. (**A**) Mice bearing bilateral subcutaneous KPC-N tumors were treated with PBS, K3-SPG-ISV (10 μg on days 9, 12, and 14), anti-CD40-ISV (20 μg on day 9), and a combination of K3-SPG-ISV/anti-CD40-ISV (same dose and schedule as respective monotherapy) (n = 4). Tumor volume of the treated side (left panel) and untreated side (center panel) and survival curves (right panel) are shown. Data are representative of two independent experiments with similar results. (**B**) Splenocytes were isolated on day 19 of the experiment under the same protocol described in panel A, stained with fluorochrome-conjugated antibodies, and subjected to flow cytometric analysis for identification of naïve, memory, effector CD8 T cells. Gating strategy is shown in Supplementary Fig. 6. Representative flow cytometry plots from two independent experiments are shown (left panel). Right panels show the absolute number of effector, memory, and naïve CD8 T cells within 10^6^ splenocytes (n = 3). (**C**) Liver metastatic model mice harboring firefly luciferase-tagged colon-26 were treated with PBS or a combination of K3-SPG-ISV (10 μg)/ anti-CD40-ISV (100 μg) on days 7, 8, 9, 10, and 11 after tumor inoculation (n = 6). Right upper panel shows the experimental design. Red vertical bars indicate the timing of bioluminescence imaging with IVIS. Left panel shows bioluminescence images, taken after 7, 11, and 14 days of implantation. Right lower panel shows the bioluminescence signal intensity of control and in situ vaccinated mice for monitoring tumor volumes. The arrows indicate the timing of therapy. Error bars represent the mean ± SEM. Statistically significant differences were measured by one-way ANOVA followed by the Tukey–Kramer test (**A**, **B**) and Dunnett’s post hoc tests (**C**). *p < 0.05; **p < 0.01. Survival curves were analyzed using the log-rank test.

The activation of CD40 on DCs through the interaction with CD40 ligand (CD40L) plays an important role in the induction of antitumor T cell responses. Thus, we reasoned that intratumoral injection of agonistic CD40 antibody (anti-CD40-ISV) might partner well with K3-SPG-ISV to achieve more potent ISV. Indeed, in the bilateral PDAC model, anti-CD40-ISV as well as K3-SPG-ISV suppressed tumor growth on both the treated and untreated sides, demonstrating the abscopal effect of each respective monotherapy. The combination of K3-SPG-ISV/anti-CD40-ISV resulted in enhanced tumor suppression and prolonged survival longer than each monotherapy (Fig. 6A). Flow cytometric analysis of splenic CD8 T cells revealed that either monotherapy with K3-SPG-ISV or anti-CD40-ISV shifted naïve (CD44- CD62L+) or memory (CD44+ CD62L+) phenotype at the baseline to effector (CD44+ CD62L-) phenotype and that the combination of K3-SPG-ISV/anti-CD40-ISV further increased the frequency of effector cells, suggesting that this ISV combination enhanced T cell priming (Fig. 6B).

Finally, the systemic antitumor effect of the K3-SPG-ISV/anti-CD40-ISV combination was evaluated using a liver metastasis model of CRC that is closer to human clinical practice. Firefly luciferase-tagged colon-26 cells were injected subcutaneously into the spleen so that tumor cells would be engrafted in the liver, followed by a combination of K3-SPG-ISV/anti-CD40-ISV. Monitoring of bioluminescence imaging of luciferase-expressing cells clearly showed that this ISV combination suppressed both the vaccinated tumor and metastatic liver lesions, further confirming the induction of systemic response by ISV (Fig. 6C).

## Discussion

As we have reported previously, K3-SPG, a second-generation TLR9 ligand, has superior properties to other previously known CpG ODNs in that it is a clinically translatable water-soluble nanoparticulate with the ability to induce robust type I IFN^9^. Accordingly, K3-SPG-iv has been demonstrated to act as a potential vaccine adjuvant for cancer and viral infection in mouse and NHP models^9,17–21^. In this study, focusing on the clinical feasibility and advantages of ISV as mentioned in the introduction section, we evaluated the mono- or combined therapies of K3-SPG-ISV using mouse models of PDAC and CRC, two representative cancer types in the fields of pancreaticobiliary and gastrointestinal oncology. Our results can be summarized as follows: K3-SPG-ISV (1) suppresses tumor growth and prolongs survival, (2) is more potent than K3-ISV or K3-SPG-iv, (3) synergizes with either systemic administration of CPIs (anti-PD-1 and anti-CTLA-4 antibodies) or intratumoral administration of agonistic-CD40 antibody, another innate immune stimulator, (4) induces, even when used as monotherapy, systemic and long-lasting memory responses, (5) induces an interferogenic immunostimulatory TME, and (6) expands effector CD8 T cells in the spleen, increases the number of tumor-infiltrating lymphocytes and suppresses tumor growth in a CD8 T cell dependent manner. The antitumor activity of K3-SPG-iv was shown to be dependent on both type I IFN, IL-12, and Batf3-positive cross-presenting DCs^21^. Therefore, although formal proof needs to be provided, we consider the same mechanisms to operate in K3-SPG-ISV. Regarding immune effectors induced by K3-SPG-ISV, the data presented herein only provide insight into the essential role of CD8 T cells. Future experiments are warranted to determine the role of other cell types including CD4 T cells.

CPIs targeting PD-1 and CTLA-4, the current standards of cancer immunotherapy, have the limitation of a low response rate^2,3^. Accordingly, most efforts in this field are directed toward developing CPI-based combinations with other classes of immunotherapy that have different mechanisms of action from CPIs^31^. The desirable effects of ISV in that the tumor itself can be turned into a vaccine and that the immunosuppressive shield is broken in the TME might increase the likelihood of response to CPIs. Indeed, there are several preclinical and clinical studies of ISV utilizing TLR9 agonists, IMO-2125 (B type), SD-101 (C type), MGN1703 (C type) and CMP-001 (D type), in combination with pembrolizumab, atezolizumab, or ipilimumab for melanoma, lung cancer, pancreatic cancer and colorectal cancer^13,32–39^. Considering the advantages of K3-SPG over C-, D-, and B-types of TLR9 agonists^9^, the synergism of K3-SPG-ISV with CPIs encourages the use of K3-SPG as an adjuvant to CPIs to improve their response rate. On the other hand, another critical limitation of CPIs is the problem of serious systemic adverse effects^40,41^. A challenging but promising strategy to overcome this shortcoming is to develop an immunotherapy that is sufficiently effective even without CPIs. The remarkable finding of this study is that K3-SPG-ISV monotherapy sufficiently induced both systemic and memory responses, the two most appealing advantages of cancer immunotherapy (Figs. 4, 5). This observation is very important because, although these immune responses have been reported to be induced in previous preclinical studies of ISV, most studies incorporated CPIs as partners of ISV^42,43^. Together with the observation that additional local innate immune stimulation by anti-CD40-ISV further enhanced the antitumor activity of K3-SPG-ISV (Fig. 6), the CPI-free ISV strategy that utilizes either K3-SPG alone or in combination with anti-CD40-ISV might be a promising option for achieving effective antitumor activity without serious systemic adverse effects caused by CPIs. In any scenario, the K3-SPG-based ISV could satisfy unmet needs in the current field of cancer immunotherapy.

In contrast to preclinical studies where the protocol of tumor puncture can be optimized to provide proof of concept, multiple repeated tumor punctures are not practical in clinical settings. Therefore, it is important to maximize the efficacy of ISV in a single puncture to minimize the number of punctures. The activation of CD40 on DCs through interaction with its cognate ligand CD40L licenses DCs to boost CD8 T cell priming^30^. Our observation that the addition of anti-CD40-ISV-enhanced K3-SPG-ISV encourages the development of ISV incorporating other classes of innate immune stimulators, such as CD40 agonists, to maximize efficacy. Besides targeting CD40, intratumoral injection of fms-related tyrosine kinase 3 ligand or granulocyte–macrophage colony-stimulating factor might also be promising options, because they recruit and activate DCs at the tumor site^44–46^. Cancer immunity is activated by ICD that releases tumor antigens from dead tumor cells and local innate immune activation, the latter of which is mainly aimed at by ISV. Thus, an alternative maximization strategy for ISV should be incorporation of local therapeutic interventions that accelerate tumor antigen release. ISV could be combined with radiotherapy^47^, photodynamic therapy^48^, thermal ablation^49^, irreversible electroporation^37,50^, cryoablation^51^ or the recently developed near-infrared photoimmunotherapy. As our previous results showed that ICD is induced by K3-SPG itself^21^, these additional interventions would synergize with K3-SPG. Our group is currently conducting studies to identify the best partner of K3-SPG-based ISV.

The high incidence of recurrence after surgical resection is problematic for patients with PDAC and CRC^52,53^. The perioperative immunotherapy would prevent tumor recurrence by overcoming postoperative immunosuppression and enhancing antitumor immunity^54^. While our results encourage the clinical application of K3-SPG-based ISV for therapeutic purposes, the vaccine strategy presented here should also operate in the context of neoadjuvant immunotherapy that utilizes the preoperative tumor as a vaccine, thereby inducing systemic and memory responses for surveying and destroying the micrometastatic lesions causing postoperative recurrence. Although a clinical trial of intratumoral injection of TLR9 ligand (CMP-001) along with nivolumab for resectable melanoma has been conducted (NCT03618641), to the best of our knowledge, published data are only available for neoadjuvant immunotherapy using only CPIs^55^. Future studies are warranted to investigate the usefulness of K3-SPG-based ISV as neoadjuvant immunotherapy.

An obvious limitation of ISV is the technical hurdle of intratumoral injection. This study is from the standpoint of emphasizing ISV, which argues against safety concerns regarding systemic therapy. Nonetheless, the therapeutic value of K3-SPG-iv is not discounted, particularly for tumors that are not accessible, because its adjuvant effect has been proven^21^, and an undesirable systemic inflammatory response has been shown to be weaker in K3-SPG-iv than in TLR3 ligand poly(I:C)-iv in NHP experiments^19^. Careful pretreatment evaluation of the accessibility of tumors and selection of patients for ISV are crucial. Our observation that K3-SPG-ISV remains effective in PDAC model which is resistant to K3-SPG-iv presumably due to insufficient intratumoral accumulation of K3-SPG (Fig. 2F) might encourage the indication of K3-SPG-ISV for refractory but accessible cancer types.

In summary, even K3-SPG-ISV monotherapy has remarkable potential for inducing both systemic and memory immune responses. The present results also demonstrated that K3-SPG-ISV synergizes with systemic administration of CPIs or local administration of agonistic CD40 antibodies. Taken together, K3-SPG-ISV can be combined with CPIs to improve their response rate or, conversely, applied as CPI-free local immunotherapy with or without an additional innate immune stimulator to avoid CPI-related adverse events. Our results provide a strong rationale for clinical translation of K3-SPG-based ISV to gastrointestinal and hepatopancreatobiliary malignancies for which endoscopy-, ultrasound-, or EUS-guided puncture is a routine clinical technique.

## Methods

This study was carried out in accordance with relevant guidelines and regulations.

### Mice

C57BL/6 and BALB/c wild-type mice were purchased from Charles River Laboratories (Yokohama, Japan). All the mice were maintained under specific pathogen-free conditions. No specific sex selection was used in this study. The protocols of all mouse experiments were approved by the Institutional Animal Care and Use Committee and the Ethics Committee of Kyoto University Graduate School of Medicine (Med Kyo 20315). All animal experiments were carried out in accordance with ARRIVE guidelines.

### Cell lines and reagents

Colon-26 cells were obtained from RIKEN (Tsukuba, Japan). Colon-26-Luc expressing firefly luciferase was obtained as a kind gift from Dr. Nikaido. MC38 was obtained as a kind gift from Dr. Honjo’s Laboratory. The PDAC cell lines KPC-N and KPF-T were established from Kras^LSL-G12D/+^, Trp53^LSL-R172H/+^, Pdx1-Cre (KPC) mice and Kras^FSF-G12D/+^, Trp53^FSF-R172H/+^, Pdx1-Flp (KPF) mice, respectively, as previously described^56,57^. Colon-26 cells were cultured in RPMI-1640 medium (FUJIFILM Wako Pure Chemical Corporation), and KPC-N, KPF-T, and MC38 were cultured in Dulbecco’s modified eagle medium (FUJIFILM Wako Pure Chemical Corporation) at 37 °C in a 5% CO_2_ incubator. Each culture medium was supplemented with 10% fetal bovine serum and 1% penicillin/streptomycin. K3 and K3-SPG were prepared as described previously^11^. Anti-mouse PD-1 antibody (clone RMP1-14), anti-mouse CTLA-4 antibody (clone 9D9), anti-mouse CD8α antibody (clone YTS 169.4), isotype control IgG (clone LTF-2), and anti-mouse CD40 antibody (clone FGK45) were purchased from Bio X Cell. All the antibodies were endotoxin-free.

### In vivo mouse studies

For the implantable tumor experiments, PDAC or CRC cells (2 × 10^6^ cells) in 100 μL of 10% Matrigel (Corning Life Sciences) in PBS were injected subcutaneously into the left or bilateral flanks of 6-to 8-week-old mice. The mice were randomized into each treatment group and treatment was started 7–10 days after tumor inoculation when palpable tumors were present (approximately 20–60 mm^3^ calculated as described below). At the indicated time points described in the figure legends, mice were injected intratumorally (it) with K3 (30 μg/100 μL), K3-SPG (10 μg/100 μL), or agonistic anti-CD40 antibody (20 μg/100 μL or 100 μg /100 μL), intravenously (iv) with K3-SPG (10 μg/100 μL), and intraperitoneally (ip) with anti-PD-1 antibody (100 μg/100 μL) or anti-CTLA-4 antibody (100 μg/100 μL). For T cell depletion, anti-CD8α antibody (200 μg/100 μL) was intraperitoneally injected twice weekly for the duration of the experiment, starting on day 0 (day of enrollment). For isotype controls, rat IgG2b (200 μg/100 μL) was used under the same protocol. Tumor length (L) and width (W) were measured at least twice weekly. The tumor volume (V) was calculated using the formula V = L × W × W/2. The endpoint criteria for survival studies included tumor volumes exceeding 4,000 mm^3^.

### In vivo bioluminescence analysis in the liver metastasis model

The liver metastasis model was generated by intrasplenic injection of 2 × 10^5^ firefly luciferase-tagged colon-26 cells and subcutaneous injection of 2 × 10^6^ cells into the right flank of the mice. The subcutaneous tumor of the mice was treated with K3-SPG (10 μg/100 μL) and anti-CD40 antibody (100 μg/100 μL) or PBS as a control at the indicated time points indicated in the figure legend. Luciferase activity was measured using the IVIS Lumina II in vivo imaging system (PerkinElmer) according to the manufacturer’s instructions.

### In vitro stimulation and cytokine measurement by ELISA

Human PBMCs obtained from healthy donors who provided informed consent were purchased from HemaCare. Human PBMCs (1 × 10^6^) cultured in 96 wells plate were stimulated in triplicate for 24 h with K3, K3-SPG, and D35 at the concentrations indicated in the figure legends. Culture supernatants were harvested and subjected to assays with human IFN-α ELISA kit (Abcam) and human IL-12p40 ELISA kit (BioLegend), according to the manufacturer’s instructions. The experiments were approved by the Ethics Committee of Kyoto University Graduate School of Medicine (R1004). To prepare mouse splenocytes, spleens were harvested from sacrificed mice and mechanically homogenized into single-cell suspensions filtered through 40-μm nylon filters into PBS with additional red blood cell (RBC) lysis using RBC Lysing Buffer Hybri-Max (Sigma-Aldrich). Mouse splenocytes (1 × 10^6^) cultured in 96 wells plate were stimulated in triplicate for 24 h with K3 and K3-SPG at the concentrations indicated in the figure legends. Culture supernatants were harvested and subjected to assays with mouse IFN-α ELISA kit (PBL Assay Science) and mouse IL-12p40 ELISA kit (R&D Systems) according to the manufacturer’s instructions.

### IFN-γ ELISPOT assay

CD8 T cells were isolated from a single-cell suspension of splenocytes prepared from KPC-N tumor-bearing mice, using CD8 + T cell Isolation Kit (Miltenyi Biotec). For in vitro stimulation, 1 × 10^5^ CD8 T cells were cocultured with 1 × 10^4^ KPC-N target cells in 96 wells plate for 24 h at 37 °C. 1 × 10^4^ MC38 cells were used as negative control to assess the target specificity. IFN-γ-producing cells were quantified using Mouse IFN-γ ELISPOT kit (R&D Systems) according to the manufacturer’s instructions. IFN-γ spots were counted on ImmunoSpot S6 Analyzer (Cellular Technology Limited).

### Immunohistochemistry

Extracted tumor tissues were fixed with 10% neutral phosphate-buffered formalin and embedded in paraffin. For immunohistochemical staining, antigen retrieval was performed by incubating the sections in citric acid buffer (pH 6.0) for 15 min at 98 °C. Then, endogenous peroxidase was quenched with 0.3% hydrogen peroxide in methanol at room temperature for 30 min. Blocking was performed by incubating the sections with a blocking solution (Dako). After blocking, the sections were incubated at 4 °C overnight with the following primary diluted antibodies: anti-CYP2E1 (dilution, 1:500; Abcam). Primary antibody incubation was performed at 4 °C overnight in a humidified chamber. The primary antibody used was a rabbit anti-CD8α antibody (dilution 1:100; Cell Signaling Technology, Cat.# 98941). Subsequently, the sections were incubated with peroxidase-labeled polymer conjugated secondary antibody (Dako) for 60 min at room temperature. Immunoreactivity was detected with diaminobenzidine substrate kit (Dako), and the sections were counterstained with hematoxylin.

### Flow cytometry

Mouse splenocytes were prepared as described above. Samples were resuspended in PBS with 1% fetal calf serum, followed by incubation with anti-CD16/32 mAb (Fc block, clone 93, BioLegend) for 10 min at 4 °C to prevent nonspecific Fc receptor binding. Samples were then stained for 40 min at 4 °C with various combinations of fluorochrome-conjugated antibodies: anti-CD45-PerCP (clone 30-F11, BioLegend), anti-CD8α-APC (clone 53–6.7, BD Biosciences), anti-CD3-Alexa Fluor 488 (clone 145-2C11, BD Biosciences), anti-CD44-PE-Cy7 (clone IM7, BioLegend), and anti-CD62L-BV421 (clone MEL-14, BioLegend). To exclude dead cells from the analysis, a fixable viability stain 510 (BD Biosciences) was used. Samples were analyzed using BD FACS Aria II and the collected data was analyzed using FlowJo software (Tree Star).

### RNA isolation and quantitative real-time PCR analysis

Total RNA was isolated from human PBMCs, mouse splenocytes, and explanted tumors using RNeasy kit (QIAGEN) according to the manufacturer’s instructions. Complementary DNA was synthesized from 500 ng of input RNA using the ReverTra Ace qPCR RT Master Mix (Toyobo) and subjected to quantitative real-time PCR (qPCR) with a SYBR Green-based gene expression assay using a LightCycler 480 System (Roche) as described previously^45^. The expression levels were standardized by comparing the levels of mouse 18S rRNA or human GAPDH as reference genes. All reactions were performed in triplicate. Primer sequences are listed in the Supplementary Table.

### Statistics

Differences between groups were analyzed by one-way analysis of variance (ANOVA) with Dunnett’s post hoc test (for two groups) or one-way ANOVA with the Tukey–Kramer test (for more than two groups). Survival curves were assessed using the log-rank test. All statistical analyses were performed using JMP Pro version 14.0.0 (SAS Institute Inc.). Differences were considered statistically significant at *p* < 0.05, and are denoted as *p < 0.05; **p < 0.01; ***p < 0.001. Error bars represent the mean ± standard error of the mean (SEM).

## Supplementary Information

Supplementary Information.
