# Supplementary material for: In situ vaccination using unique TLR9 ligand K3-SPG induces long-lasting systemic immune response and synergizes with systemic and local immunotherapy
Source: Sci Rep. 2022 Feb 8;12:2132. doi: 10.1038/s41598-022-05702-0 (PMC8825851; doi:10.1038/s41598-022-05702-0)
Supplement: Supplementary file 1 — Supplementary Information. [file 41598_2022_5702_MOESM1_ESM.pdf]

Supplementary Information

In situ vaccination using unique TLR9 ligand K3-SPG induces long-lasting systemic immune response and synergizes with systemic and local immunotherapy

Hirokazu Okada, Ken Takahashi, Hiroaki Yaku, Kouji Kobiyama, Keiko Iwaisako, Xiangdong Zhao, Masahiro Shiokawa, Norimitsu Uza, Yuzo Kodama, Ken J. Ishii, and Hiroshi Seno

- Supplementary Figure Legends
- Supplementary Figure1
- Supplementary Figure2
- Supplementary Figure3
- Supplementary Figure4
- Supplementary Figure5
- Supplementary Figure6
- Supplementary Table

Supplementary Figure Legends

Supplementary Figure 1. ***In vitro*** **mouse splenocyte stimulation with K3-SPG.**

A. Production of IFN-α and IL-12p40 by mouse splenocytes stimulated with K3 (0.56, 1.67, and 5 μg/mL) and K3-SPG (0.56, 1.67, and 5 μg/mL) for 24 h was measured by ELISA. B. The relative expression levels of *Ifna*, *Ifnb*, *Ifng*, *Mx1*, *Isg56*, *Rantes*, *Il12*, *Cd80*, *Cd86*, *Il6*, and *Tnfa* mRNA induced by K3 (5 μg/mL) or K3-SPG (1.67 μg/mL) were measured by quantitative real-time PCR. The results were normalized to the expression of 18S rRNA. Data are representative of two independent experiments with similar results. Error bars represent the mean ± SEM. *$p<0.05$, **$p<0.01$, ***$p<0.001$; statistically significant differences were measured by one-way ANOVA followed by the Tukey–Kramer test.

Supplementary Figure 2. Antitumor activity of K3-SPG-ISV in another PDAC mouse model.

Tumor volume (left panel) and survival rate (right panel) were monitored in KPF-T-bearing mice treated with PBS or K3-SPG-ISV (10 μg) on days 10, 13, 16, 19, and 24 after tumor inoculation (n=5). The arrows indicate the timing of therapy. Error bars represent the mean ± SEM. Statistically significant differences were measured by one-

way ANOVA with Dunnett’s post hoc test. **p<0.01, ***p<0.001. Survival curves were analyzed using log-rank tests.

Supplementary Figure 3. Antitumor activity of K3-SPG-ISV potentiates the effect of CTLA-4 blockade therapy.

The left panel shows the tumor volume of KPC-N-bearing mice treated with PBS, K3-SPG-ISV (10 μg on days 9, 11, and 14), anti-CTLA-4-ip (100 μg on days 9, 11, and 14), or a combination of K3-SPG-ISV/anti-CTLA-4-ip (same dose and schedule as respective monotherapy) (n=4). The right panel shows the tumor volume of colon-26-bearing mice treated with PBS, K3-SPG-ISV (10 μg on days 9, 11, 14, 16, and 18), anti-CTLA-4-ip (100 μg on days 9, 11, and 14), or a combination of K3-SPG-ISV/anti-CTLA-4-ip (same dose and schedule as respective monotherapy) (n=5). The arrows indicate the timing of therapy. Error bars represent the mean ± SEM. Statistically significant differences were measured using one-way ANOVA followed by the Tukey–Kramer test. *p<0.05. ip, intraperitoneal

Supplementary Figure 4. Immunological memory induced by the combination of K3-SPG-ISV/anti-PD-1 in CRC model.

Colon-26-bearing mice were treated with PBS (n=4) or a combination of K3-SPG-ISV (10 μg on days 7, 9, 12, 14, and 16) and anti-PD-1-ip (100 μg on days 5, 7, and 9) (n=6). Blue and green arrows indicate the timing of K3-SPG-ISV and anti-PD-1-ip, respectively. Three out of six K3-SPG-ISV/anti-PD-1-ip-treated mice were cured. On day 105, the cured mice were rechallenged with the second round of subcutaneous inoculation of 2×10^6 colon-26 cells and age-matched naïve BALB/c mice (n=5) were subcutaneously inoculated with the same number of colon-26 as a control cohort. The tumor volume of each mouse is presented.

Supplementary Figure 5. K3-SPG-ISV induces systemic antitumor effects in a CRC model that is dependent on CD8 T cells.

A. Mice bearing bilateral subcutaneous colon-26 tumors were treated with PBS or K3-SPG-ISV (10 μg) at one tumor site on days 9, 11, 14, 17, and 19 after tumor inoculation (n=5). Tumor volumes on the treated side (left panel) and untreated side (right panel) are shown. B. Colon-26-bearing mice were treated with PBS/isotype control, K3-SPG-ISV (10 μg)/isotype control, PBS/anti-CD8α, or K3-SPG-ISV (10 μg)/anti-CD8α (n=5). K3-SPG-ISV was performed on days 9, 11, 14, 16, and 18 after tumor inoculation. Anti-CD8α was administered twice weekly for the duration of the experiment, starting on day 0. The

left panel shows the tumor volume of each cohort. The right panel shows the flow cytometry plots demonstrating CD8 T cell depletion in splenocytes on day 30. The arrows indicate the timing of therapy. Error bars represent the mean ± SEM. Statistically significant differences were measured by one-way ANOVA with Dunnett's post hoc test. *$p<0.05$. CD8 dep: CD8 depletion by anti-CD8α

Supplementary Figure 6. Gating strategy for FACS analysis.

The gating strategies for identifying effector CD8 T cells (CD45+, CD3+, CD8+, CD44+, and CD62L-), memory CD8 T cells (CD45+, CD3+, CD8+, CD44+, and CD62L+), and naïve CD8 T cells (CD45+, CD3+, CD8+, CD44-, and CD62L+) are shown.

Supplementary Figure 1

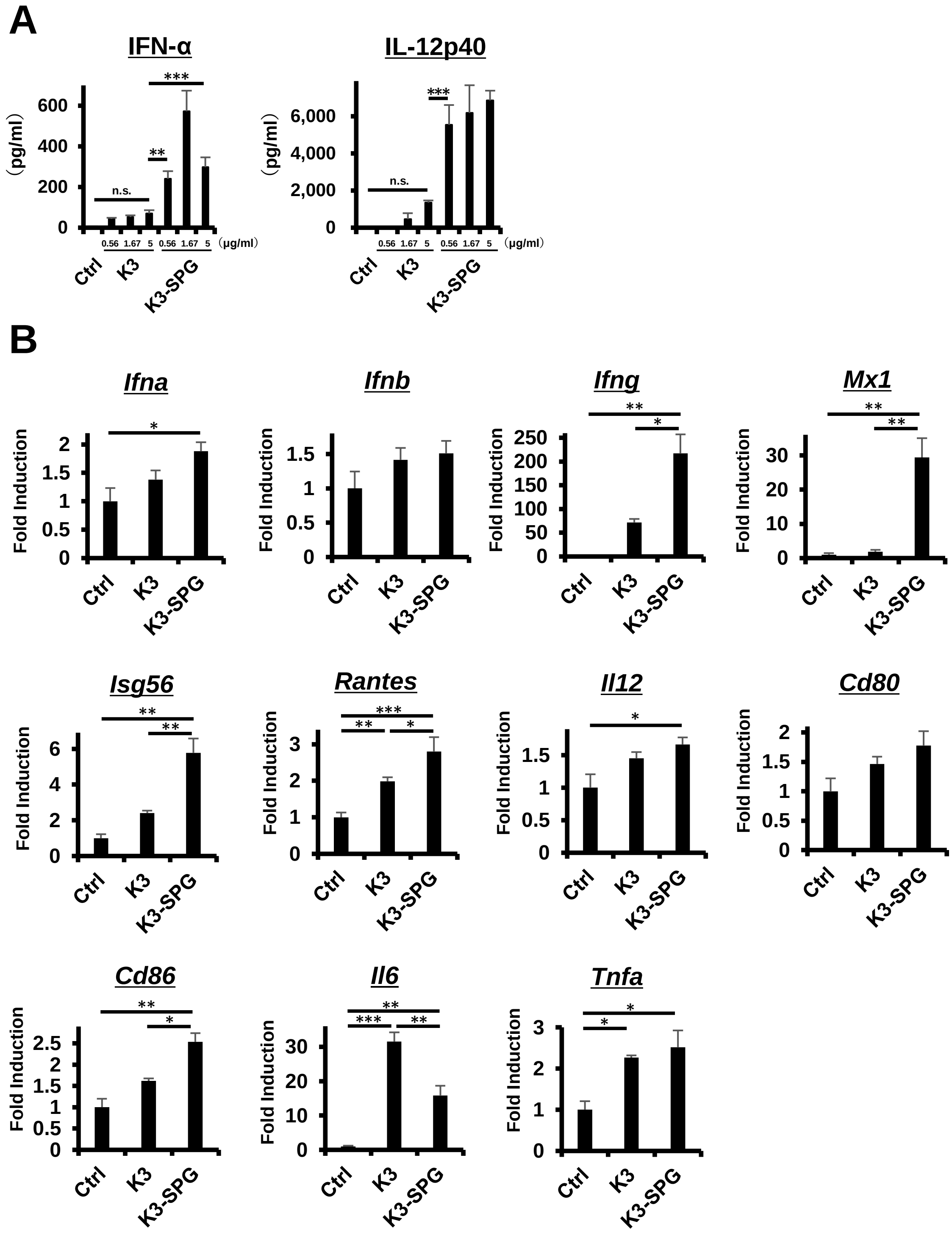

Supplementary Figure 2

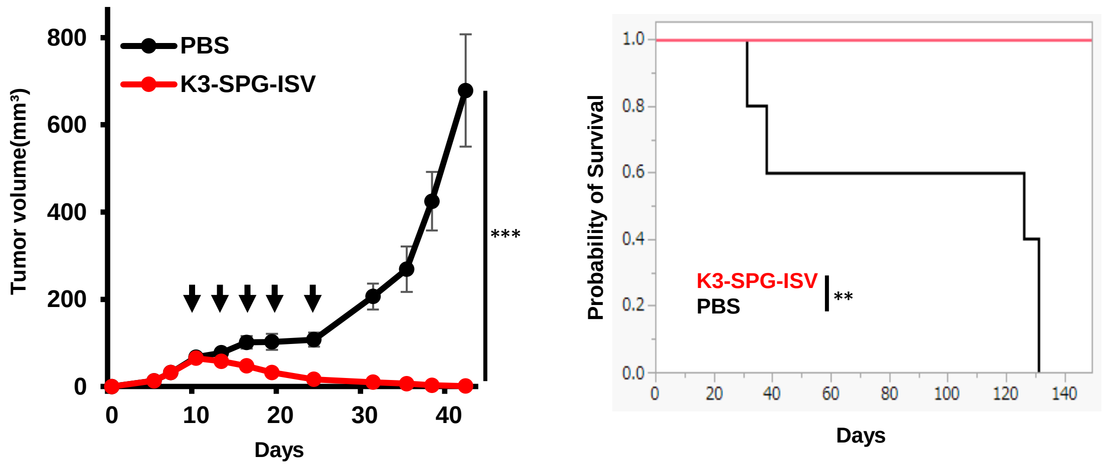

Supplementary Figure 3

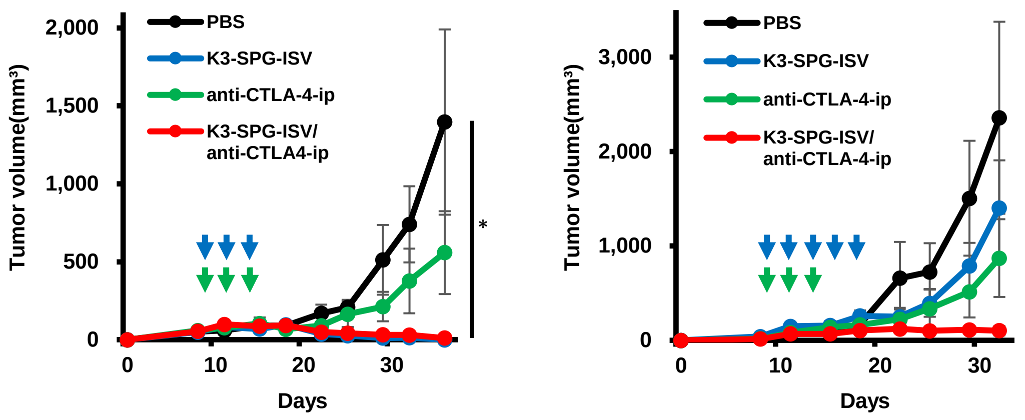

Supplementary Figure 4

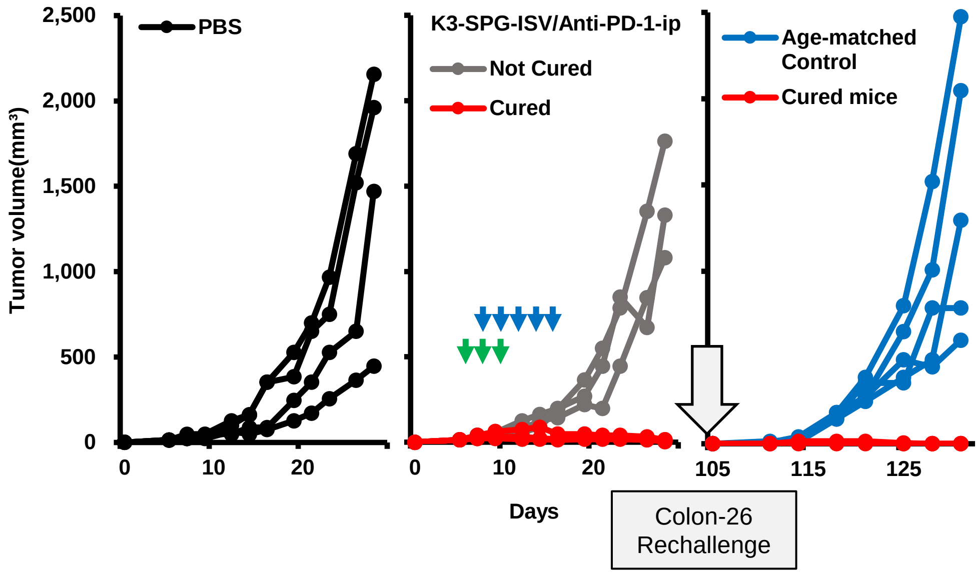

Supplementary Figure 5

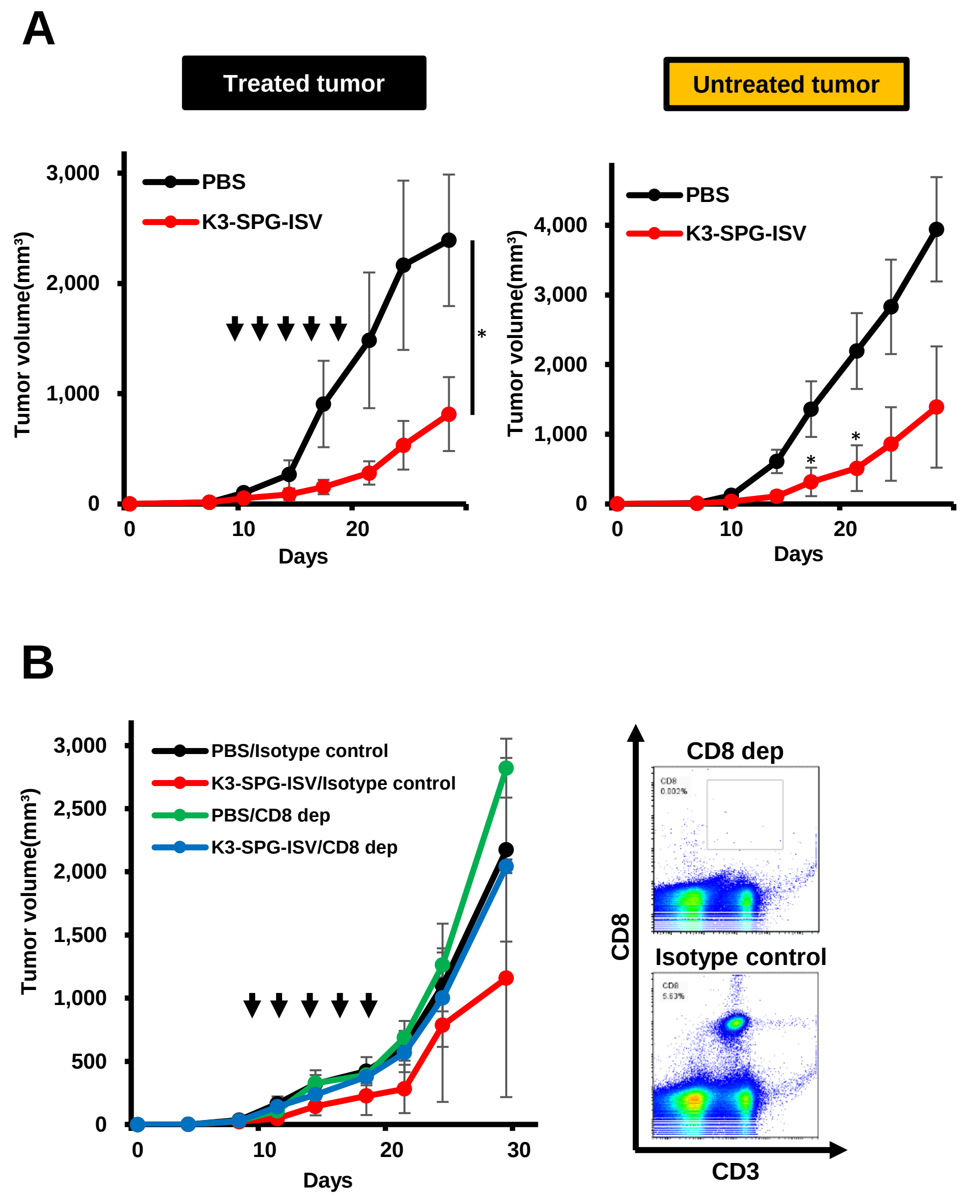

Supplementary Figure 6

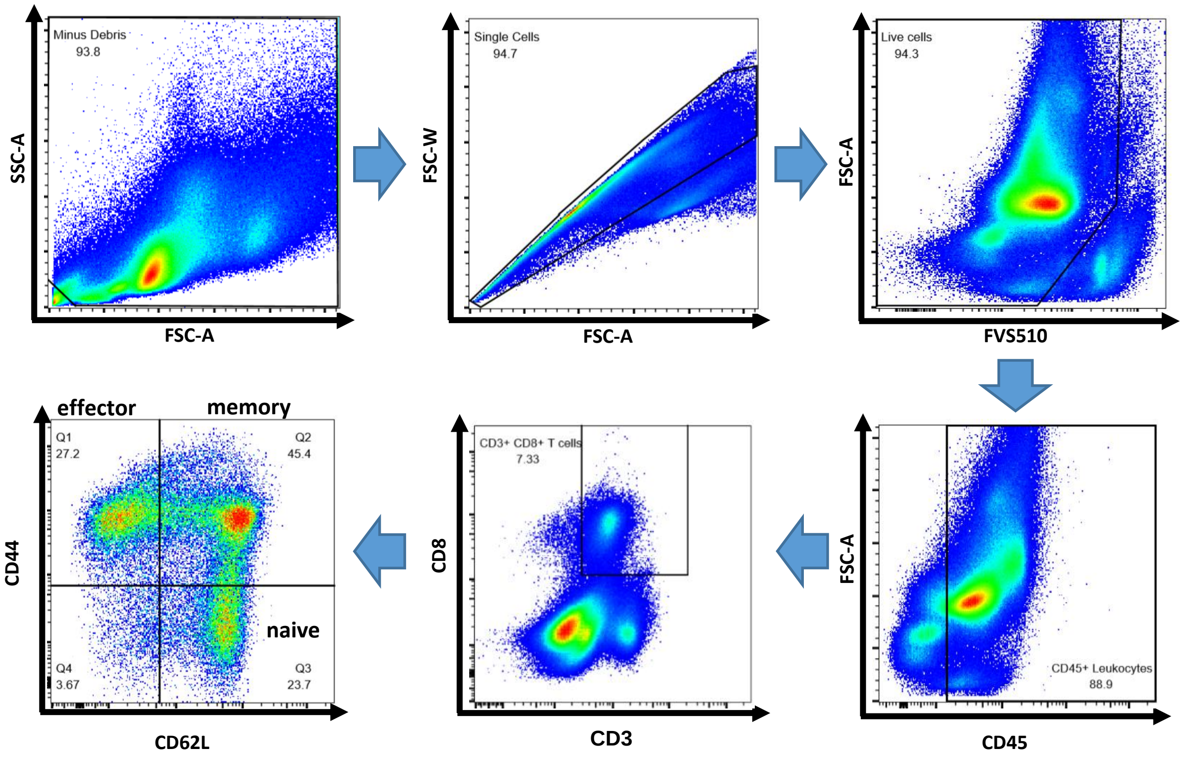

Supplementary Table. List of primer sequences used for real-time PCR.

**Species**	**Gene**	**Forward Primer (5'- ＞ -3')**	**Reverse Primer (5'- ＞ -3')**
Human	*GAPDH*	GAAGGTGAAGGTCGGAGTC	GAAGATGGTGATGGGATTTC
	IFNA	TAGGCTCACCCATTTCAACCAG	CAGGAGGGCCACCAGTAAAG
	IFNB	GACATCCCTGAGGAGATTAAGCA	CAACAATAGTCTCATTCCAGCCA
	IFNG	CCAACGCAAAGCAATACATGA	CGCTTCCCTGTTTTAGCTGC
	MXA	ACAGGACCATCGGAATCTTG	CCCTTCTTCAGGTGGAACAC
	RANTES	CATCTGCCTCCCCATATTCCT	AGTGGGCGGGCAATGTAG
	IL12	CCTGCTGGTGGCTGACGACAAT	CTTCAGCTGCAAGTTGTTGGGT
	CD80	TGGTGCTGGCTGGTCTTTC	CTGTGCCACTTCTTTCACTTCC
	CD86	ACATTCTCTTTGTGATGGCCTTC	TGCAGTCTCATTGAAATAAGCTTGA
	IL6	CCAGCTATGAACTCCTTCTC	GCTTGTTCCTCACATCTCTC
	TNFA	GAAAGCATGATCCGGGACGTG	GATGGCAGAGAGGAGGTTGAC
Mouse	*18S rRNA*	GTAACCCGTTGAACCCCATT	CCATCCAATCGGTAGTAGCG
	Ifna	TGCAACCCTCCTAGACTCATTCT	CCAGCAGGGCGTCTTCCT
	Ifnb	AGCTCCAAGAAAGGACGAACAT	GCCCTGTAGGTGAGGTTGATCT
	Ifng	TGAACGCTACACACTGCATCTTGG	CGACTCCTTTTCCGCTTCCTGAG
	Mx1	GACTACCACTGAGATGACCC	CTCTATTTCCTCCCCAAATG
	Isg56	GGGCCTTGCAGGCATCACCTT	TCCTGCCTTCTGGGCTGCCT
	Rantes	GCCCACGTCAAGGAGTATTTCTA	ACACACTTGGCGGTTCCTTC
	Il12	ACTCTGCGCCAGAAACCTC	CACCCTGTTGATGGTCACGAC
	Cd80	TTTTCAGGTTGTGAAACTCAACCTT	TCCACCCGGCAGATGCTA
	Cd86	TGTTTCCGTGGAGACGCAAG	CAGCTCACTCAGGCTTATGTTTT
	Il6	TCCAGTTGCCTTCTTGGGAC	GTACTCCAGAAGACCAGAGG
	Tnfa	GGCATGGATCTCAAAGACAACC	CAGGTATATGGGCTCATACCAG
